# Oxidative damage and myofiber degeneration in the gastrocnemius of patients with peripheral arterial disease

**DOI:** 10.1186/1479-5876-11-230

**Published:** 2013-09-25

**Authors:** Dustin J Weiss, George P Casale, Panagiotis Koutakis, Aikaterini A Nella, Stanley A Swanson, Zhen Zhu, Dimitrios Miserlis, Jason M Johanning, Iraklis I Pipinos

**Affiliations:** 1 Department of Surgery, University of Nebraska Medical Center, Omaha, NE 68198-5182, USA; 2 Department of Surgery, Omaha Veterans Affairs Medical Center, Omaha, NE, USA

## Abstract

Peripheral arterial disease (PAD), a manifestation of systemic atherosclerosis that produces blockages in arteries supplying the legs, affects an estimated 27 million people in Europe and North America. Increased production of reactive oxygen species by dysfunctional mitochondria in leg muscles of PAD patients is viewed as a key mechanism of initiation and progression of the disease. Previous studies demonstrated increased oxidative damage in homogenates of biopsy specimens from PAD gastrocnemius compared to controls, but did not address myofiber-specific damage. In this study, we investigated oxidative damage to myofibers as a possible cause of the myopathy of PAD. To achieve this, we developed and validated fluorescence microscopy procedures for quantitative analysis of carbonyl groups and 4-hydroxy-2-nonenal (HNE) adducts in myofibers of biopsy specimens from human gastrocnemius. PAD and control specimens were evaluated for differences in 1) myofiber content of these two forms of oxidative damage and 2) myofiber cross-sectional area. Furthermore, oxidative damage to PAD myofibers was tested for associations with clinical stage of disease, degree of ischemia in the affected leg, and myofiber cross-sectional area. Carbonyl groups and HNE adducts were increased 30% (p < 0.0001) and 40% (p < 0.0001), respectively, in the myofibers of PAD (N = 34) compared to control (N = 21) patients. Mean cross-sectional area of PAD myofibers was reduced 29.3% compared to controls (p < 0.0003). Both forms of oxidative damage increased with clinical stage of disease, blood flow limitation in the ischemic leg, and reduced myofiber cross-sectional area. The data establish oxidative damage to myofibers as a possible cause of PAD myopathy.

## Introduction

Peripheral arterial disease (PAD) is a manifestation of atherosclerosis that produces progressive narrowing and occlusion of arteries supplying the lower extremities. In Europe and North America, the prevalence of PAD is estimated at 16% of individuals 55 years and older, which corresponds to 27 million people, 10.5 million of whom are symptomatic [1,2]. The majority of PAD patients experience claudication, *i.e.,* walking-induced leg muscle pain relieved by rest, and their disease is classified as Fontaine Stage 2 [3]. In the later stages of PAD, patients experience foot pain at rest (Fontaine Stage 3) and/or non-healing ulcers, necrosis and gangrene (Fontaine Stage 4). Although the primary problem in PAD patients is the presence of atherosclerotic blockages in the arteries supplying their legs [4-6], altered, arterial hemodynamics is not the only cause of functional limitation in the lower limbs of PAD patients [7-12].

Several laboratories including our own have demonstrated that a myopathy is present in the legs of patients with PAD [4-6]. This myopathy is characterized by progressive myofiber degeneration with fibrous and/or fatty deposition [13,14] and a defect in mitochondrial energy metabolism [15-17] characterized by reduced activities of mitochondrial electron transport chain complexes in association with increased carbonyl and 4-hydroxy-2-nonenal (HNE) damage to whole muscle protein [11]. However, the precise relationship between oxidative damage and the myopathy of PAD remains to be determined. Assuming that oxidative damage to myofibers is a principal cause of the myopathy of PAD, we hypothesized that mean oxidative damage per myofiber increases with advancing disease, in association with declining myofiber cross-sectional area. We tested this hypothesis by quantitatively comparing oxidative damage within the myofibers of biopsy specimens from PAD and control gastrocnemius, and by testing myofiber oxidative damage for associations with Fontaine stage, hemodynamic limitation of the PAD limb and myofiber cross-sectional area. This rigorously quantitative, observational approach is essential for designing pre-clinical studies that are driven by specific histological, cellular and molecular features of the disease and, therefore, offer improved translational performance [18].

## Materials and methods

### Human subjects

The Institutional Review Boards of the VA Nebraska-Western Iowa Medical Center and University of Nebraska Medical Center approved the experimental protocol and all subjects gave informed consent.

#### PAD group

We recruited 34 consecutive patients undergoing lower extremity operations for symptomatic PAD (Table 1). For every patient, the diagnosis of PAD was based on medical history, physical examination, significantly decreased ankle-brachial index (ABI < 0.9) and computerized or standard arteriography demonstrating stenoses and/or occlusions in the arteries supplying the lower extremities. The diagnostic workup revealed evidence of aortoiliac disease alone in three patients, femoropopliteal disease alone in seven patients, aortoiliac and femoropopliteal disease in 11 patients, aortoiliac and femoropopliteal and crural occlusive disease in five patients and femoropopliteal and crural disease in eight patients. PAD in patients presenting with intermittent claudication and no symptoms of ischemic rest pain and no evidence of tissue loss was classified as Fontaine stage 2. PAD in patients presenting with ischemic rest pain and no evidence of tissue loss was classified as stage 3. PAD in patients presenting with ischemic, non-healing ulcers and/or gangrene was classified as stage 4. Seven patients underwent aortobifemoral bypass grafting, eight patients underwent femoropopliteal bypass grafting, seven patients underwent femorotibial bypass grafting, three patients underwent combined aortofemoral and femoropopliteal bypass grafting and the other nine patients underwent a major amputation procedure.

**Table 1:** Demographics of PAD and control patients

	**Control**	**PAD**	**p-value**
**Number of subjects**	21	34	N/A
Mean Age (years)*	64.0 ± 9.3	61.5 ± 7.4	0.278
Height (m)*	1.79 ± 0.10	1.76 ± 0.06	0.175
Weight (kg)*	91.2 ± 14	81.6 ± 19	0.057
BMI*	28.9 ± 4.3	26.4 ± 6.2	0.117
**Gender (male/female)**	19/2	32/2	0.868
**Smoking (%)**	52.4	73.5	0.109
**Coronary Artery Disease (%)**	23.8	61.7	0.006
**PCI**^**§ **^**(%)**	23.8	14.7	0.480
**CABG**^**‖ **^**(%)**	14.3	14.7	0.966
**Myocardial Infraction (%)**	19.0	20.6	0.890
**Stroke (%)**	4.7	3.0	0.726
**Obesity**^**¶ **^**(%)**	19	23.5	0.695
**Dyslipidemia (%)**	47.6	55.8	0.551
**Diabetes mellitus (%)**	23.8	29.4	0.650
**Hypertension (%)**	57.1	82.3	0.041
**Renal Insufficiency**^**† **^**(%)**	14.3	8.80	0.528
**Ankle brachial index**^**‡**^	1.13 ± 0.21 (0.94-1.34)	0.34 ± 0.24 (0.01-0.81)	< 0.001

*Data are presented as mean ± SD.

^**¶**^Obesity: Body mass index higher than 30.

§PCI = percutaneous coronary intervention.

‖CABG = coronary artery bypass graft.

†Renal insufficiency: estimated creatinine clearance less than 60 ml/min/1.73 m^2^.

‡Data are presented as mean ± SD and (minimum-maximum value).

#### Control group

We recruited 21 patients with normal blood flow to their lower limbs, undergoing lower extremity operations for indications other than PAD (Table 1). These patients had no history of PAD symptoms, and all had normal lower extremity pulses at examination. All controls had normal ABIs at rest and after stress and all led sedentary lifestyles.

#### Biopsy

Gastrocnemius samples weighing approximately 250 mg were obtained from the anteromedial aspect of the muscle belly, 10 cm distal to the tibial tuberosity. All biopsy specimens were obtained with a 6 mm Bergstrom needle. Some samples were frozen for biochemical analysis and some were placed immediately into cold methacarn. After 48 hours in methacarn, the specimens were transferred to cold ethanol: H_2_O (50:50 v/v) and subsequently embedded in paraffin.

### Quantitative fluorescence microscopy

#### Quantitative labeling of hydrazide-reactive carbonyl groups and 4-hydroxy-2-nonenal (HNE) adducts in gastrocnemius specimens

**Figure 1:**
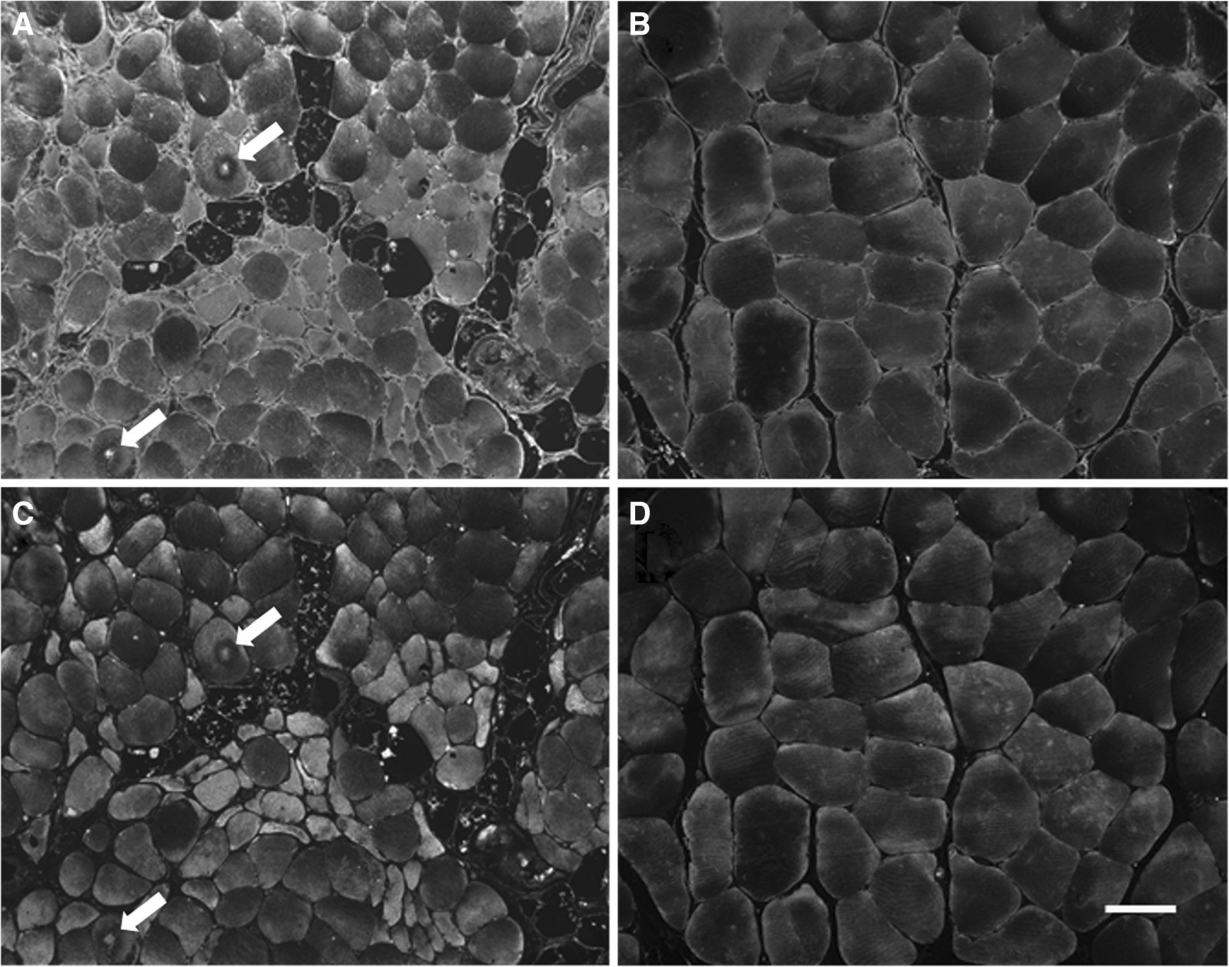
**Oxidative damage determined as carbonyl groups (Panels A and B) and HNE adducts (Panels C and D), is increased in gastrocnemius myofibers of patients with peripheral arterial disease (Panels A and C) compared to the control patients (Panels B and D).** The control muscle has polygonal myofibers of similar shape and size. The PAD muscle exhibits a wide range of myofiber sizes with a smaller average myofiber size. Additionally, the PAD muscle has fatty infiltration, endomysial fibrosis (increased extracellular matrix between myofibers) and target lesions with evidence of increased oxidative damage (arrows). Levels of oxidative damage varied widely among PAD myofibers but similar patterns of injury were seen with carbonyl and HNE labeling. Oxidative damage in PAD muscle was not limited to the myofiber compartment but was consistently elevated throughout the extracellular matrix where it was present exclusively as carbonyl groups. HNE adducts were confined to the interior of the myofibers and were not detected in the extracellular matrix. Specimens obtained by needle biopsy of the gastrocnemius were fixed in cold methacarn, embedded in paraffin, sectioned at 4 μ and mounted to glass slides. Carbonyl groups in slide-mounted needle biopsy specimens were labeled with biocytin hydrazide plus streptavidin-Alexa Fluor® 488 **(**Panels **A** and **B)** and HNE adducts were labeled with monoclonal anti-HNE antibody plus goat anti-mouse IgG-Alexa Fluor® 568 **(**Panels **C** and **D)**. Images of each microscopic field were captured with a 10X objective. The white bar represents a length of 50 microns.

Paraffin-embedded biopsy specimens sectioned at 4 microns were labeled with three fluorescent reagents for quantification of ROS-induced oxidative damage in myofibers. These procedures are presented in detail in “Additional file 1” and are briefly described as follows. Carbonyl groups [19,20] and the Michael adduct of HNE [21] were labeled (Figure 1) with procedures that permitted non-overlapping measurement of these distinct forms of oxidative damage. As a means of partitioning individual myofibers for measurement of oxidative damage, sarcolemmas were labeled with Wheat Germ Agglutinin. For measurement of protein carbonyls [22,23], endogenous biotin groups were blocked and then carbonyl groups were biotinylated by treatment of slide specimens with 5 mM biocytin-hydrazide (EZ-Link Hydrazide-Biocytin, product # 28020; Thermo Scientific-Pierce Protein Research Products, Rockford, IL, USA). For measurement of HNE adducts [21], specimens were treated with a mouse monoclonal antibody (20 ug/mL) (product # MAB3249; R&D Systems, Minneapolis, MN, USA) specific for the Michael adduct of HNE. Control slides were treated with IgG2b κ isotype control (20 ug/mL) from non-immunized mice (product # 14-4732-82; eBioscience, San Diego, CA, USA). After overnight incubation at 5°C, the slides were labeled at room temperature, with a mixture of Alexa Fluor® 488 conjugated streptavidin (10 ug/mL) (product # S11223; Life Technologies-Molecular Probes, Eugene, OR, USA) and Alexa Fluor® 568 conjugated goat anti-mouse IgG (10 ug/mL) (product # S11223;Life Technologies-Molecular Probes, Eugene, OR, USA). Myofiber sarcolemmas were labeled with Alexa Fluor® 350 conjugated Wheat Germ Agglutinin (10 ug/mL) (product # W11263; Life Technologies-Molecular Probes, Eugene, OR, USA). The specimens were mounted in ProLong® Gold anti-fade medium with DAPI nuclear stain (product # P36931; Life Technologies-Molecular Probes, Eugene, OR, USA).

#### Image acquisition and analysis

Quantification of carbonyl groups and HNE adducts within individual myofibers and measurement of myofiber cross-sectional area, were based on three-channel imaging [24,25] of each microscopic field. Fluorescence images were captured with the 10x objective of a widefield, epifluorescence microscope (Leica DMRXA2; North Central Instruments, Plymouth, MN, USA) and a B/W CCD camera (Orca ER C4742-95; Hamamatsu Photonics, Bridgewater, NJ, USA), with Image-Pro® Plus software (Media Cybernetics, Bethesda, MD, USA). Each of 5 to 15 microscopic fields per specimen (400 to 2800 myofibers) was captured in three fluorescence channels corresponding to 1) myofiber sarcolemma, 2) carbonyl groups and 3) HNE adducts. Fluorescence signal produced by carbonyl groups or HNE adducts within each myofiber was expressed as mean pixel intensity in grayscale units (gsu) (on a 12-bit gray scale), which corresponds to concentration within the myofiber. Fluorescence signal was corrected for background (typically near the black level of the camera) and the mean of all myofibers in each specimen was determined. Quantification of the fluorescence signals produced by carbonyl groups and HNE adducts was validated by linear correlation of both signals across specimens obtained from the PAD patients (N = 34) (Additional file 1: Figure S1) and by Reverse-Phase Protein Array analysis (Additional file 1: Figure S2, A and B).

#### Analysis of myofiber cross-sectional area and oxidative damage in PAD and control gastrocnemius specimens

For each PAD and control patient, quartiles of oxidative damage (carbonyl or HNE signal) were determined to evaluate the association of myofiber cross-sectional area and oxidative damage. The median, upper and lower quartiles of oxidative damage for both the carbonyl and HNE signals were determined and defined four classes (Q1, Q2, Q3 and Q4) of myofibers. Q1 includes all fibers at or below the lower quartile. Q2 includes all fibers at or below the median and above the lower quartile. Q3 includes all fibers at or below the upper quartile and above the median. Q4 includes all fibers above the upper quartile. The mean of each class was determined for each patient.

### Statistics

Baseline characteristics of PAD and controls subjects were compared using general linear models for continuous variables and chi-square tests for categorical variables. Categorical variables that were different between the two groups were used as covariates in subsequent analyses. Differences in myofiber cross-sectional area and content of carbonyl groups and HNE adducts in PAD compared control muscle were evaluated by analysis of covariance with adjustments for CAD and HTN. The relationships of stage of disease and myofiber content of carbonyl groups and HNE adducts were evaluated by linear regression with adjustments for CAD and HTN. The relationships of myofiber content of carbonyl groups and HNE adducts to ABI were evaluated by a Pearson partial correlation with adjustments for CAD and HTN.

Changes in myofiber cross-sectional area in relation to changes in content of carbonyl groups and HNE adducts were evaluated by quartile analysis with a repeated measures model. A least-squares *post hoc* analysis was done when a significant effect was found. All analyses were implemented with SAS statistical software version 9.3 (SAS Institute Inc., Cary, North Carolina, USA). Data are presented as mean and standard deviation unless stated otherwise and significance was set at p < 0.05.

## Results

The demographic information for both the PAD and control subjects is presented in Table 1. Only CAD (*χ*^2^ = 7.50, *p* = 0.006) and HTN (*χ*^2^ = 4.15, *p* = 0.041) were significantly different between the PAD and control subjects.

### Gastrocnemius specimens of PAD patients exhibited increased oxidative damage and reduced myofiber cross-sectional area

Myofibers of PAD patients exhibited a wide range of carbonyl and HNE damage and, overall, a greater burden of oxidative damage compared to myofibers of control patients (Figure 1). Carbonyl groups and HNE adducts were increased 30% (F_3,51_ = 23.15; p < 0.0001) and 40% (F_3,51_ = 14.3; p < 0.0001) respectively, in myofibers of PAD patients (n = 34) compared to controls (n = 21), after adjusting for CAD and HTN (Table 2). In biological systems, HNE adducts are present predominantly as the Michael adduct [26,27], which lacks a reactive carbonyl group, thus, fluorescence signals from carbonyl groups and the HNE adduct are non-overlapping. This non-overlapping character is mirrored in the distinctive labeling of the extra-myofiber compartment (Figure 1). For both PAD and control specimens, HNE labeling was localized to the interior of the myofibers while substantial carbonyl labeling was observed within myofibers as well as in the extra-myofiber compartment. Intra-myofiber abundances of both biomarkers exhibited agreement a) within individual specimens, as seen by a fiber-by-fiber comparison of their labeling intensities (Figure 1) and b) across specimens obtained from the PAD patients (N = 34), as determined by correlation analysis of the mean values of the specimens (Additional file 1: Figure S1). The mean cross-sectional area of PAD myofibers was reduced 29.3% compared to controls (F_3,51_ = 7.65; p < 0.0003) after adjusting for CAD and HTN, suggesting that increased oxidative damage produced loss of structural integrity of PAD myofibers (Table 2).

**Table 2:** Oxidative damage and cross-sectional area of myofibers from PAD and control patients

	**Controls (n = 21)**	**PAD (n = 34)**	**p-value**
Carbonyl Content (gsu*) (mean ± SD)	486 ± 135	695 ± 132	< 0.0001
**HNE Content (gsu) (mean ± SD)**	261 ± 101	436 ± 119	< 0.0001
**Cross-Sectional Area**^**‡**^**(mean ± SD)**	5,324 ± 1,371	3,760 ± 1,546	0.0003

*Grayscale units (12-bit gray scale) of background-corrected fluorescence emission from labeled carbonyl groups and HNE adducts.

^‡^Cross-sectional area is given in square microns (μ^2^).

### Oxidative damage in gastrocnemius myofibers was associated with disease stage and ankle brachial index

**Figure 2:**
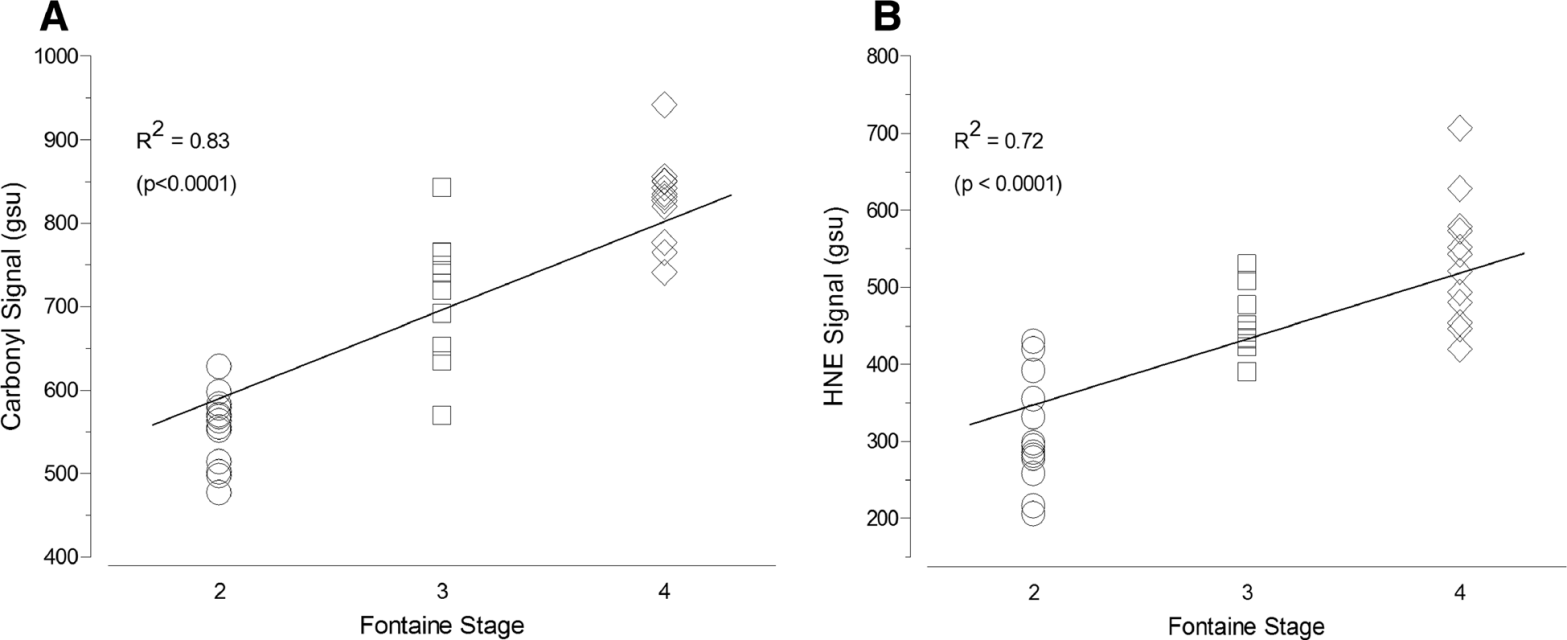
**Scatterplots of carbonyl (Panel A) and HNE (Panel B) damage as a function of Fontaine Stage of disease.** PAD patients (n = 34) were assigned a disease level of ‘2’ (N = 13), ‘3’ (N = 9), or ‘4’ (N = 12) corresponding to Fontaine Stage 2 (claudication), Stage 3 (rest pain) or Stage 4 (tissue loss), respectively.

**Figure 3:**
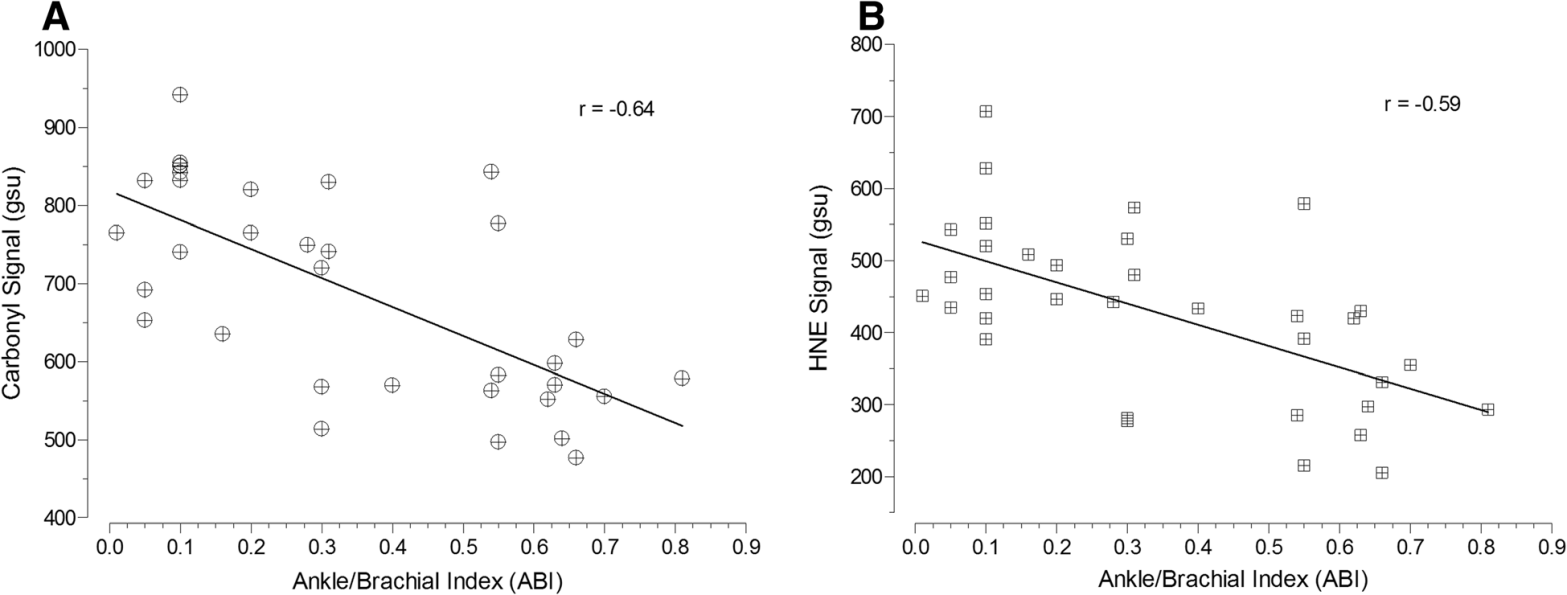
Scatterplots of carbonyl (Panel A) and HNE (Panel B) damage as a function of the Ankle Brachial Index (ABI).

The extent of oxidative damage as expressed by content of carbonyl groups and HNE adducts in gastrocnemius myofibers of PAD patients was associated with the patient’s stage of disease, adjusting for CAD and HTN (Figure 2). PAD patients (n = 34) were assigned a disease level of ‘2’ (N = 13), ‘3’ (N = 9), or ‘4’ (N = 12) corresponding to Fontaine Stage 2 (claudication), Stage 3 (rest pain) or Stage 4 (tissue loss), respectively [3]. Carbonyl content increased (R^2^ = 0.83, p < 0.0001; Figure 2A) through advancing stage of disease: Fontaine Stage 2 (552 ± 43 gsu), Stage 3 (707 ± 80 gsu) and Stage 4 (827 ± 132 gsu). Similarly, HNE content increased (R^2^ = 0.72, p < 0.0001; Figure 2B) through advancing stage of disease: Fontaine Stage 2 (310 ± 71 gsu), Stage 3 (454 ± 44 gsu) and Stage 4 (548 ± 82 gsu). In agreement with these findings, myofiber carbonyl content was inversely correlated (r = −0.64, 95% CI: [−0.80 to −0.38], p < 0.0001) with ABI (Figure 3A). Similarly, HNE content in the gastrocnemius myofibers was inversely correlated (r = −0.59, 95% CI: [−0.77 to −0.31], p < 0.001) with ABI (Figure 3B).

### The observed increase of oxidative damage in PAD gastrocnemius was associated with a significant decrease of myofiber cross-sectional area

Cross-sectional area of myofibers in PAD muscle decreased significantly in association with increased oxidative damage defined by quartiles of carbonyl (p < 0.001) or HNE (p < 0.001) content. Specifically, for carbonyl damage the cross-sectional area of Q4 myofibers was significantly reduced when compared to Q1 (p = 0.0031), Q2 (p = 0.0012) and Q3 (p = 0.0047; Table 3) myofibers. For HNE damage the cross-sectional area of Q4 myofibers was significantly reduced when compared to Q1 (p = 0.0013), Q2 (p = 0.0019) and Q3 (p = 0.0084; Table 3) myofibers. The mean cross-sectional area of myofibers in control muscle remained the same across quartile-based groupings of carbonyl or HNE content (Table 3).

**Table 3:** Association of myofiber cross-sectional area and oxidative damage, for PAD and control patients

	**Q1**	**Q2**	**Q3**	**Q4**
**PAD myofiber area by carbonyl (μ**^**2**^**)**	3,965 ± 1778^‡^	3,931 ± 1,6320	3,788 ± 1,539	3,376 ± 1,422*
**Control myofiber area by carbonyl (μ**^**2**^**)**	5,074 ± 1,696	5,382 ± 1,521	5,396 ± 1,568	5,442 ± 1,387
**PAD myofiber area by HNE (μ**^**2**^**)**	3,945 ± 1,329	3,921 ± 1,486	3,832 ± 1,621	3,320 ± 1,568*
**Control myofiber area by HNE (μ**^**2**^**)**	5,243 ± 1,402	5,389 ± 1,260	5,304 ± 999	5,364 ± 925

^‡^Cross-sectional area in square microns (μ^2^) is presented as mean ± SD.

*Q4 is significantly different from Q1, Q2 and Q3 at p < 0.01 (for carbonyl groups and HNE adducts).

Myofibers were distributed on the basis of quartiles of carbonyl or HNE content into the following groups:

Q1: All fibers at or below the lower quartile.

Q2: All fibers at or below the median and above the lower quartileQ3: All fibers at or below the upper quartile and above the median.

Q4: All fibers above the upper quartile.

## Discussion

The present work is an extension of previous studies that established the presence of a myopathy characterized by myofiber degeneration, mitochondrial dysfunction and oxidative damage in the gastrocnemius of patients with PAD. Abnormalities in mitochondrial ultrastructure [13] and inefficient utilization of acyl-CoAs [28] constitute the earliest evidence of a mitochondriopathy in PAD muscle. Later studies established a defect in mitochondrial energy metabolism in the gastrocnemius of PAD patients with moderate claudication [15-17]. This finding together with the observation of increased ROS biomarkers in the plasma of PAD patients after a single bout of exercise-induced claudication [29,30] suggests that damaged electron transport chain complexes and increased ROS production may contribute to the pathophysiology of PAD. This conclusion is supported by a study [11] that demonstrated reduced enzymatic and respiratory activities of electron transport chain complexes I, III, and IV in PAD compared to control gastrocnemius. These changes were associated with significant increases in both carbonyl groups and HNE adducts in whole muscle protein. Similar findings were reported for a mouse model of hind limb ischemia [31], where inflow arterial occlusion alone, *i.e.,* in the absence of comorbid conditions, caused myopathy with myofiber degeneration, mitochondrial dysfunction and oxidative damage. Although our previous studies of PAD patients clearly demonstrated increased oxidative damage in ischemic muscle, they could not identify the myofiber as a site of damage. In addition, the study design we used in our previous work did not address the relationship between oxidative damage and severity of clinical disease, degree of hemodynamic limitation in the ischemic leg or myofiber cross-sectional area.

We developed a unique method based on fluorescence microscopy, for quantitative analysis of myofiber morphology and biomolecules and their chemical modifications in individual myofibers of clinical specimens taken from patients [24,25]. Oxidative damage was increased in myofibers of the gastrocnemius of PAD compared to control patients, in association with reduced myofiber cross-sectional area, suggesting that myofiber degeneration may be a consequence of the accumulation of oxidative damage within the fibers. Mean cross-sectional area of the more damaged myofibers in PAD muscle was smaller, again linking myofiber degeneration with increased oxidative damage to the myofibers. Furthermore, our data revealed that the extent of oxidative damage within myofibers of PAD muscle was associated with both limb ABI (representing hemodynamic limitation) and Fontaine Stage (representing clinical progression of the disease).

We quantified two forms of ROS-mediated oxidative damage, carbonyl groups and the Michael adduct of HNE, with procedures that detected each form exclusively of the other. Carbonyl groups (free aldehydes and ketones) are present largely as carbonylated proteins that cannot be repaired by the cell [19,20] and, consequently, accumulate at a rate dependent upon rates of carbonylation, ROS detoxification, proteolysis and the formation of insoluble oxidized protein aggregates [20]. Protein carbonyls, widely used as biomarkers of oxidative stress, represent a spectrum of oxidative modifications including direct oxidation of protein, lipid peroxidation, and protein glycation [20]. The second form of oxidative damage that we quantified was the Michael adduct of HNE. Among reactive aldehydes produced by lipid peroxidation, HNE is the most abundant and toxic of the α,β-unsaturated aldehydes [32], is formed exclusively by lipid peroxidation, and is considered to be a major contributor to the cytopathological effects of oxidative stress [33]. The primary mechanism of HNE toxicity is protein adduction rather than depletion of cellular reducing equivalents, and HNE adducts are predominantly Michael adducts [26]. The pathophysiologic importance of HNE is supported by increased tissue abundance of the aldehyde and its Michael adduct in diseases characterized by increased oxidative damage; including Alzheimer’s Disease, Parkinson’s Disease, atherosclerosis, cardiovascular disease, and chronic obstructive pulmonary disease [20].

Oxidative damage in PAD muscle was not limited to the myofiber compartment but occurred throughout the ECM, as well. Levels of oxidative damage varied widely among PAD myofibers but were consistently elevated throughout the ECM. Oxidative damage to the ECM was detected exclusively as carbonyl groups. HNE adducts were confined to the interior of the myofibers and were not detected in the ECM. These observations suggest differences in the mechanisms producing oxidative damage in the ECM and myofibers. For example, oxidative damage to the ECM may be due largely to the formation of advanced glycation end products. On the other hand, oxidative damage within the myofiber may be due largely to lipid peroxidation end products produced primarily by damaged and dysfunctional mitochondria.

An important question emerging from our findings is related to the effects of exercise on the muscles of PAD patients. On the one hand, it is widely held that exercise therapy for claudicating patients is a primary treatment option (IA rating) given the documented health improvements that follow exercise therapy [3]. On the other hand, it is possible that exercise (at least some forms of exercise or too much exercise) may not help PAD patients and may even worsen the damage in their legs. ROS biomarkers are increased in the plasma of PAD patients, after a single bout of exercise-induced claudication [29,30]. Damaged electron transport chain complexes and increased ROS production appear to be central to the pathophysiology of PAD [4-6,17,34]. Is it possible that “non-optimized” exercise induces metabolic, oxidative and inflammatory stress in the already damaged and impaired PAD limb thereby worsening the condition of the ischemic muscle [35]? The most thorough attempt to address this question was a study of claudicating patients undergoing a 12-week program of exercise therapy [36]. The study demonstrated significantly increased peak exercise performance and peak oxygen consumption. However, evaluation of gastrocnemius biopsy specimens demonstrated that training was associated with skeletal muscle injury characterized by denervation of muscle fibers (identified as a significant increase in target lesions and angular fibers) and a trend for increased connective tissue. Interestingly, a concurrent control group undergoing strength training exhibited no such changes in their muscles. The authors concluded that, over the 12 weeks of treatment, the observed changes in skeletal muscle were not of sufficient magnitude to prevent improved performance in response to the training program but that increased walking activity over time may be injurious to skeletal muscle fibers [36]. Although the benefits of exercise for the functional status of claudicating patients have been well-documented, unanswered questions remain in regard to the mechanisms underlying the effects of exercise and the potential of non-optimized exercise to produce deleterious effects in the ischemic muscle [35]. There is a need for studies that will establish the optimal prescription of exercise for the claudicating patient by carefully evaluating the effects of exercise on PAD skeletal muscle and how they relate to changes in performance.

To our knowledge, this study is the first to quantify oxidative damage to myofibers in the gastrocnemius of PAD patients, on a fiber-by-fiber basis, and to relate these changes to clinical stage of disease, severity of arterial occlusions and myofiber cross-sectional area. Importantly, we have established that fixed, paraffin-embedded biopsy specimens may be used to quantify molecular changes in the affected muscles of PAD patients. This methodology can produce quantitative profiles of disease- and stage-specific molecular pathologies of PAD. This may help us to: 1) develop highly representative models of the disease in animals or cell culture, 2) follow and possibly predict the natural history of PAD in a particular limb and patient, 3) design and direct individualized, targeted treatment for the PAD patient, and 4) establish a reliable biomarker signature for PAD-related myofiber degeneration that can be used for direct evaluation of clinical interventions, *e.g.,* exercise and revascularization. Oxidative damage to the muscular system of the lower limbs appears to be of central importance in the pathogenesis of PAD and more work is needed to improve our knowledge of oxidative stress mechanisms in the legs of PAD patients.

The principal limitation of our study is that it did not identify cause and effect linkages between carbonyl groups or HNE adducts and PAD. Instead, the study demonstrated that each form of oxidative damage was increased within myofibers and was associated with both disease progression and worsening ABIs. Consequently, this study identified oxidative damage to myofibers as a potential mechanism of PAD. This quantitative, observational approach is essential to designing hypotheses that are highly relevant to human disease and, therefore, support the design of more effective translational studies [18].

## Conclusions

In summary, our work demonstrated increased oxidative damage to gastrocnemius myofibers in PAD patients that was associated with increased hemodynamic impairment, reduced myofiber cross-sectional area and disease stage. These findings are consistent with myofiber oxidative damage as a significant contributor to the pathophysiology of PAD. They also provide insight into the chronic and progressive nature of PAD, where accumulation of oxidative damage in the ischemic limb may cause patients to transition from claudication to critical limb ischemia and eventually limb loss. Finally, our quantitative molecular profiling of human biopsy specimens may be a potential tool for designing effective translational studies and targeted interventions needed for improved diagnosis, staging and treatment of PAD.

## Abbreviations

PAD: Peripheral arterial disease; ABI: Ankle brachial index; HNE: 4-hydroxy-2-nonenal; ROS: Reactive oxygen species; BMI: Body mass index; CAD: Coronary artery disease; HTN: Hypertension; PCI: Percutaneous coronary intervention; CABG: Coronary artery bypass graft; ECM: Extracellular matrix; SD: Standard deviation.

## Competing interests

The authors declare that they have no competing interests.

## Authors’ contributions

DJW carried out the immunofluorescence analysis, data collection and image analysis, and drafted the manuscript. GPC designed the methodology and critically revised the manuscript. PK carried out the statistical analysis and image analysis. AAN participated in the design of the study and image analysis. SAS prepared the specimens for analysis and participated in the design. ZZ prepared the specimens for analysis and participated in the design. DM participated in its design and coordination. JMJ participated in the design, coordination and drafting the manuscript. IP conceived of the study, participated in its design and critically reviewing the manuscript. All authors read and approved the final manuscript.

## Supplementary Material

Additional file 1: Figure S1 Correlation of mean carbonyl signal per myofiber with the mean HNE signal, across the 34 PAD patients. **Figure S2**. Validation of Quantitative Fluorescence Microscopic (QFM) analysis of carbonyl groups **(A)** and the HNE adducts **(B)** in slide-mounted gastrocnemius specimens, by Reverse-Phase Protein Array (RPPA) analysis of the corresponding frozen tissue homogenates. **Figure S3**. Reproducible quantification of carbonyl groups and HNE adducts in gastrocnemius myofibers, across analytical sessions. Click here for file
