# Supplementary material for: Oxidative damage and myofiber degeneration in the gastrocnemius of patients with peripheral arterial disease
Source: J Transl Med. 2013 Sep 25;11:230. doi: 10.1186/1479-5876-11-230 (PMC3849592; doi:10.1186/1479-5876-11-230)
Supplement: Additional file 1: Figure S1 — Correlation of mean carbonyl signal per myofiber with the mean HNE signal, across the 34 PAD patients. Figure S2. Validation of Quantitative Fluorescence Microscopic (QFM) analysis of carbonyl groups (A) and the HNE adducts (B) in slide-mounted gastrocnemius specimens, by Reverse-Phase Protein Array (RPPA) analysis of the corresponding frozen tissue homogenates. Figure S3. Reproducible quantification of carbonyl groups and HNE adducts in gastrocnemius myofibers, across analytical sessions. [file 1479-5876-11-230-S1.docx]

**Additional File 1**

**Materials and Methods**

*Quantitative labeling of hydrazide-reactive carbonyl groups and 4-hydroxy-2-nonenal (HNE) adducts in gastrocnemius specimens:* Slide-mounted needle biopsy specimens of the gastrocnemius were deparaffinized, rehydrated, and treated with 100 mM citrate buffer (pH 6.0) at 95^o^ C for 10 min. For measurement of protein carbonyls [[1](#_ENREF_1), [2](#_ENREF_2)], endogenous biotin was blocked and then carbonyl groups were biotinylated by treatment of slide specimens, first with 5 mM biocytin-hydrazide (EZ-Link Hydrazide-Biocytin, product # 28020; Thermo Scientific-Pierce Protein Research Products, Rockford, IL, USA) in 100 mM 2-morpholinoethane sulfonic acid buffer (pH 6.5) (product # M3671-250G; Sigma-Aldrich, St. Louis, MO, USA) for 1 hr and then with 15 mM cyanoborohydride (product # 156159-10G; Sigma-Aldrich, St. Louis, MO, USA) in Dulbecco’s PBS at pH 7.2 (product # 14190-367; Life Technologies-Invitrogen, Grand Island, NY, USA) for 30 min. For measurement of HNE adducts [[3](#_ENREF_3)], specimens were blocked with 10% goat serum (product # 50062Z; Life Technologies-Invitrogen, Grand Island, NY, USA) and treated with a mouse monoclonal antibody (20 ug/mL) (product # MAB3249; R&D Systems, Minneapolis, MN, USA) specific for the Michael adduct of HNE. Control slides were treated with IgG (20 ug/mL) from non-immunized mice (product # 14-4732-82; eBioscience, San Diego, CA, USA). After overnight incubation at 5^o^ C, the slides were labeled for 1 hr, at room temperature, with a mixture of Alexa Fluor® 488 conjugated streptavidin (10 ug/mL) (product # S11223; Life Technologies-Molecular Probes, Eugene, OR, USA) and Alexa Fluor® 568 conjugated goat anti-mouse IgG (10 ug/mL) (product # S11223; Life Technologies-Molecular Probes, Eugene, OR, USA). The conditions for both carbonyl and HNE labeling were adjusted to achieve maximal fluorescence signals required for quantitative analysis [[4](#_ENREF_4), [5](#_ENREF_5)]. Subsequently, myofiber sarcolemmas were labeled for 1 hr with Alexa Fluor® 350 conjugated Wheat Germ Agglutinin (10 ug/mL) (product # W11263; Life Technologies-Molecular Probes, Eugene, OR, USA). The specimens were mounted in ProLong Gold® anti-fade medium with DAPI nuclear stain (product # P36931; Life Technologies-Molecular Probes, Eugene, OR, USA). Duplicate slides of each biopsy specimen were analyzed. Control slides exhibited no detectable fluorescence labeling. Additionally, carbonyl labeling decreased >80% in specimens pretreated with 0.10 M sodium borohydride (product # 480886-25G; Sigma-Aldrich, St. Louis, MO, USA) to reduce carbonyl groups to non-reactive alcohol groups. Similarly, HNE labeling was decreased >90% with 4 x 10 ^-5^ M HNE-N-acetylhistidine [[6](#_ENREF_6), [7](#_ENREF_7)] present in the primary antibody solution.

Validation of the use of wide-field fluorescence microscopy for quantification of carbonyl groups and HNE adducts in tissue sections: To establish our microscopy-based analyses of carbonyl groups and HNE adducts as quantitative measurements rather than semi-quantitative classifications, we completed both an internal and an external validation of our measurements. First, as an internal validation, we established that the mean content of carbonyl groups per myofiber correlated directly (r=0.84; p<0.001) with the mean HNE content across the 34 PAD patients in this study **(Figure S1)**. Second, as an external validation, we established that the microscopy-based measurements of carbonyl groups and HNE adducts correlated directly with measurements obtained by an independent analysis of the corresponding muscle homogenates **(Figure S2)**. Tissue sections and muscle homogenates prepared from biopsy specimens of seven patients, representing a broad range of oxidative damage were analyzed by fluorescence microscopy and reverse-phase protein array analysis (RPPA) [[5](#_ENREF_5)], respectively. Frozen muscle (140 mg/patient) was homogenized in buffer containing protease inhibitor cocktail (product # P8340; Sigma-Aldrich, St. Louis, MO, USA) and centrifuged and then the supernatant was analyzed by RPPA as described previously [[4](#_ENREF_4)]. For analysis of carbonyl groups, 70 uL of each supernatant and an oxidized BSA standard were treated with an equal volume of 1.0 mM 2,4-dinitrophenylhydrazine (product # 42210; Sigma-Aldrich, St. Louis, MO, USA) in phosphoric acid (adjusted to pH 6.2) for one hr at room temperature. The reaction was stopped by addition of 60 uL of a solution (pH 7.0) consisting of 2 M Tris, 30% (v/v) glycerol and 1.3 uL 2-mercaptoethanol (product # M7522; Sigma-Aldrich, St. Louis, MO, USA), and serial two-fold dilutions of supernatant and standard were spotted in triplicate to nitrocellulose slides (Oncyte Avid Nitrocellulose Film-Slides, product # 305170; Grace Bio-Labs, Bend, OR, USA). The slides were probed with rabbit polyclonal anti-DNP antibody (2 ug/mL) (product # 713500; Life Technologies-Molecular Probes, Eugene, Or, USA) and labeled with IRDye 800cw-conjugated goat anti-rabbit IgG (0.4 ug/mL) (product # 926-32211; LI-COR Biosciences, Lincoln, NE, USA). For analysis of HNE adducts, serial two-fold dilutions of supernatant and HNE-BSA standard were spotted in triplicate to nitrocellulose slides. Slides were probed with mouse monoclonal anti-HNE antibody (2 ug/mL) (product # MAB3249; R&D Systems, Minneapolis, MN, USA) and labeled with IRDye 800cw-conjugated goat anti-mouse IgG (0.4 ug/mL) (product # 926-32210; LI-COR Biosciences, Lincoln, NE, USA). Integrated fluorescence of each spot, determined with the LI-COR Odyssey system (LI-COR Biosciences, Lincoln, NE, USA), was corrected for background produced by rabbit IgG (product # 02-6102; Life Technologies-Molecular Probes, Eugene, OR, USA) or mouse IgG (eBioscience, San Diego, CA, USA) and the means for triplicate spots were computed. Plots of fluorescence intensities of the homogenates against mean pixel intensities of the corresponding slide specimens had R^2^ values of 0.86 (p=0.014) and 0.93 (p=0.003) for carbonyl groups (**Figure S2A**) and HNE adducts (**Figure S2B**), respectively, validating our microscopy-based procedure.

Quality control for unbiased, reproducible analysis of ROS-induced oxidative damage in gastrocnemius myofibers: Every analytical session included a mixture of control and PAD biopsy specimens, each represented by two slide specimens. Slide specimens were labeled with a robotic autostainer (BioGenex *i*6000, BioGenex Laboratories Inc., Freemont, CA, USA). All analytical sessions subsequent to the first run included three control and three PAD specimens analyzed in the previous session. At the start of each image acquisition session, excitation lamp energy was evaluated with fluorescent, calibration beads (InSpeck Calibration Kit, 6 μ beads, product # I14785; Life Technologies-Molecular Probes, Eugene, OR, USA). These procedures established highly reproducible measurements within and across analytical sessions as determined by statistical analysis of the Intraclass Correlation Coefficient (ICC)[[8](#_ENREF_8)]. Intrasession and intersession reliability were estimated by using a two-way mixed ANOVA with absolute agreement. An ICC of less than 0.40 indicates poor reproducibility, while an ICC between 0.40 and 0.75 indicates fair to good reproducibility. Lastly, an ICC that is greater than 0.75 indicates excellent reproducibility. Intrasession reliability was estimated by the averages of the duplicate slides for 14 control and 20 PAD specimens. For the set of duplicate, control slides, the average absolute difference of each slide from the mean of its duplicate pair was 3% for carbonyl groups and 2% for HNE adducts. The corresponding ICC reliability values for the control specimens were 0.89 for carbonyl groups and 0.96 for HNE adducts. For the set of PAD slides, the average difference was 2% for carbonyl groups and 4% for HNE adducts. The corresponding ICC reliability values for the PAD specimens were 0.90 for carbonyl groups and 0.85 for HNE adducts. Across-session reproducibility was also high for both carbonyl groups and HNE adducts. Intersession reproducibility was estimated from the averages for multiple control and PAD biopsy specimens, analyzed a second time in the next analytical session. The two-session mean of each biopsy specimen was determined and each session mean was expressed as the absolute difference from the two-session mean. A typical set of three control and three PAD biopsy specimens (**Figure S3**) yielded an average difference of 9 % for carbonyl groups and 9 % for HNE adducts. The corresponding ICC reliability values for the control specimens were 0.69 for carbonyl groups and 0.77 for HNE adducts, and for the PAD specimens the ICC values were 0.76 for carbonyl groups and 0.98 for HNE adducts. The ICC values and per cent differences from the mean indicate good to excellent reproducibility of our measurements.

**Figure S1.** Correlation of mean carbonyl signal per myofiber with the mean HNE signal, across the 34 PAD patients

**Figure S2.** Validation of Quantitative Fluorescence Microscopic (QFM) analysis of carbonyl groups (A) and the HNE adducts (B) in slide-mounted gastrocnemius specimens, by Reverse-Phase Protein Array (RPPA) analysis of the corresponding frozen tissue homogenates.

**Figure S3.** Reproducible quantification of carbonyl groups and HNE adducts in gastrocnemius myofibers, across analytical sessions


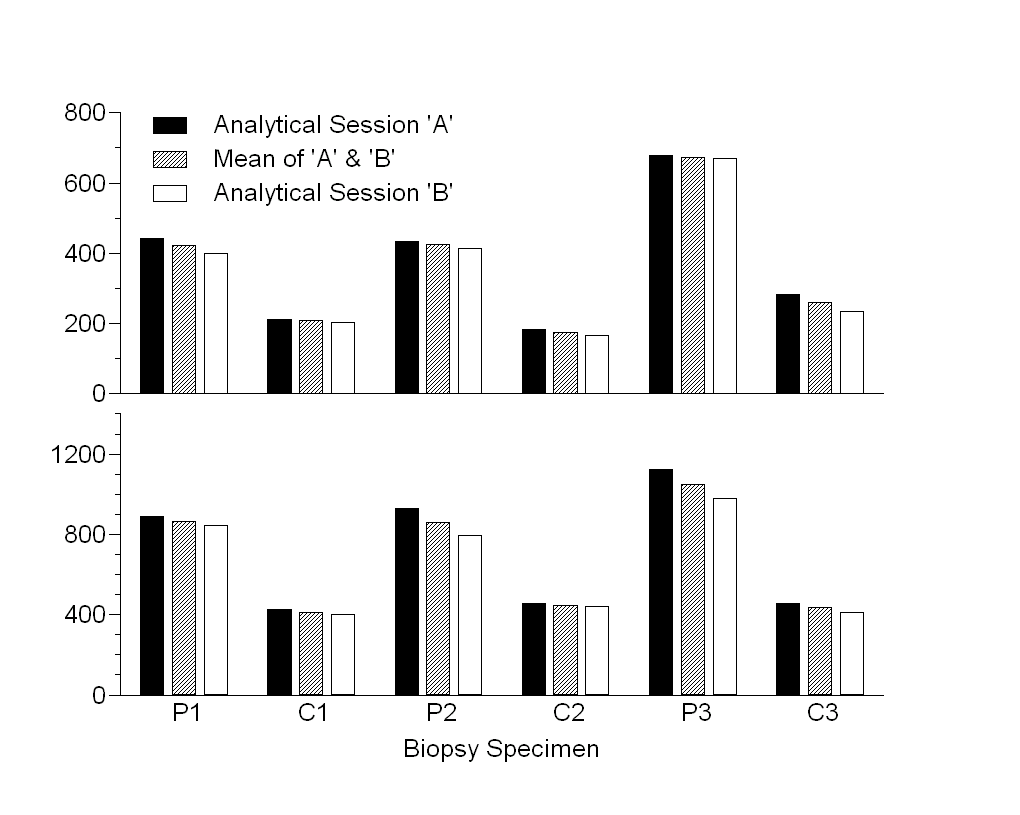


Carbonyl Groups

(Gray Scale Units)

HNE Adducts

**References:**

[1] Harris, M. E.; Carney, J. M.; Hua, D. H.; Leedle, R. A. Detection of oxidation products in individual neurons by fluorescence microscopy. *Exp Neurol* **129:**95-102; 1994.

[2] Hirao, T.; Takahashi, M. Carbonylation of cornified envelopes in the stratum corneum. *FEBS Lett* **579:**6870-6874; 2005.

[3] Uchida, K. Protein-Bound 4-Hydroxy-2-Nonenal as a Marker of Oxidative Stress. *J Clin Biochem Nutr* **36:**1-10; 2005.

[4] Huang, D.; Casale, G. P.; Tian, J.; Lele, S. M.; Pisarev, V. M.; Simpson, M. A.; Hemstreet, G. P., 3rd. Udp-glucose dehydrogenase as a novel field-specific candidate biomarker of prostate cancer. *Int J Cancer* **126:**315-327; 2009.

[5] Huang, D.; Casale, G. P.; Tian, J.; Wehbi, N. K.; Abrahams, N. A.; Kaleem, Z.; Smith, L. M.; Johansson, S. L.; Elkahwaji, J. E.; Hemstreet, G. P., 3rd. Quantitative fluorescence imaging analysis for cancer biomarker discovery: application to beta-catenin in archived prostate specimens. *Cancer Epidemiol Biomarkers Prev* **16:**1371-1381; 2007.

[6] Uchida, K.; Itakura, K.; Kawakishi, S.; Hiai, H.; Toyokuni, S.; Stadtman, E. R. Characterization of epitopes recognized by 4-hydroxy-2-nonenal specific antibodies. *Arch Biochem Biophys* **324:**241-248; 1995.

[7] Toyokuni, S.; Miyake, N.; Hiai, H.; Hagiwara, M.; Kawakishi, S.; Osawa, T.; Uchida, K. The monoclonal antibody specific for the 4-hydroxy-2-nonenal histidine adduct. *FEBS Lett* **359:**189-191; 1995.

[8] Shrout, P. E.; Fleiss, J. L. Intraclass correlations: uses in assessing rater reliability. *Psychol Bull* **86:**420-428; 1979.
